# SLEEPING MEDICATIONS AND PREVENTION OF FUNCTIONAL DISABILITY IN OLDER ADULTS WITH LOW CARE NEEDS: THE SOHA STUDY

**DOI:** 10.1093/geroni/igae098.2464

**Published:** 2024-12-31

**Authors:** Naoko Otsuki, Ryohei Yamamoto, Ayumi Kono

**Affiliations:** Osaka University, Suita, Osaka, Japan; Osaka University, Suita, Osaka, Japan; Osaka Metropolitan University, Osaka, Osaka, Japan

## Abstract

Although sleeping medications are extensively used to treat sleep disorders in older adults, their safety and efficacy in term of physical influences are controversial. A previous review reported that the risk of falls and hip fractures was associated not only with sleeping medications but also with sleep disorders. Improving sleep disorders may be vital for preventing functional disabilities. The present prospective cohort study aims to clarify the association between sleeping medication use and the incidence of functional disability in 1,652 older adults aged ≥65 years with low care needs in Japan’s long-term care insurance (LTCI). The present study included 1,291 participants aged 65 years or older at the baseline date after excluding 302 participants who received long-term care certification during the six-month baseline period. The outcome was the identification of functional disabilities based on LTCI certification. The association between the sleeping medications and the incidence of functional disabilities was assessed using Fine and Gray proportional sub-distribution hazards models. During the median observational period of 2.5 years, the incidence of functional disabilities was observed in 54.6% and 49.7% participants in the “No use” group and the “Use” group, respectively. This study clarified that the group using sleeping medications had a significantly lower risk of functional disabilities than the group not using them. (Sub-hazard ratios of “No use” and “Use”: 1.00 [reference], 0.82[CIs, 0.68–0.99]). Our findings differ from those of previous studies and may provide useful suggestions for home care services for older adults with low care needs.

